# Dogs’ recognition of human selfish and generous attitudes requires little but critical experience with people

**DOI:** 10.1371/journal.pone.0185696

**Published:** 2017-10-18

**Authors:** Fabricio Carballo, Esteban Freidin, Emma B. Casanave, Mariana Bentosela

**Affiliations:** 1 Grupo de Investigación del comportamiento en cánidos (ICOC), Instituto de Investigaciones Médicas (IDIM-CONICET), Buenos Aires, Argentina; 2 Instituto de Ciencias Biológicas y Biomédicas del Sur (INBIOSUR), Departamento de Biología Bioquímica y Farmacia, Universidad Nacional del Sur (UNS)- Consejo Nacional de Investigaciones Científicas y Técnicas (CONICET), Bahía Blanca, Argentina; 3 Instituto de Investigaciones Económicas y Sociales del Sur (IIESS; CONICET-UNS), Bahía Blanca, Argentina; Institute of Animal Science, CZECH REPUBLIC

## Abstract

There is some dispute regarding the role of experience in the development of dogs´ socio-cognitive abilities in their interaction with people. We sought to provide new evidence to this debate by comparing dogs with contrasting levels of experience with humans, in a task involving the discrimination of human generous and selfish attitudes. To this end, we compared the performance of adult family dogs against that of adult shelter dogs and puppies living in people´s homes. In training trials, the generous experimenter (G) signaled the bowl with food and allowed the dog to eat, whereas the selfish experimenter (S) also signaled the baited bowl, but she/he ate the food before the dog could have access to it. Then, subjects were allowed to freely choose between G and S in the choice test. The main finding was that adult subjects (both family and shelter dogs) developed a preference for G over S, but puppies did not. We conclude that the quality and/or quantity of everyday-contact with people did not affect the discrimination of human attitudes in the present protocol, but the amount of experience with people (in years) did matter. Finally, we discuss the relative role of domestication and ontogeny in the development of dogs´ socio-cognitive abilities.

## Introduction

Domestic dogs display sophisticated social skills in their interaction with people [1]. For instance, dogs have the capacity to discriminate between generous and selfish people, i.e., between an experimenter who facilitates access to food and an experimenter who denies its access. Experimental evidence shows that they prefer to approach the generous over the selfish person, not only after direct exchanges with them [2], but also after just observing third-party interactions [3]. This preference is presumed to be the consequence of the animal’s ability to recognize a behavioral disposition (in this case, generosity or selfishness) and associate it with a particular individual [4–5]. This capacity to track someone´s reputation is thought to have played an important role in the evolution of cooperation [6].

There are different theoretical perspectives regarding the origin of dogs´ socio-cognitive abilities. On one hand, some authors highlight the fact that dogs and people have shared the same ecological niche, and probably many similar socio-ecological problems, during the last 15,000–33,000 years [7–9]. This co-existence could have led to the convergent evolution of similar socio-cognitive abilities in dogs and humans [9–11]. This hypothesis is mainly based on evidence showing that: 1) dogs can follow human cues from very early in their ontogeny (e.g., [12, 13]); 2) dogs with scarce contact with people (e.g., shelter dogs) use human social cues similarly to dogs that live in people´s homes as pets [14]; and 3) domestic dogs show superior performance in many communicative tasks with humans relative to their closest phylogenetic relative, the wolf [13]. From this perspective, dogs would not need any prolonged exposure to people to develop the necessary abilities to use human communicative cues [13].

On the other hand, some authors stress the role of ontogenetic experience and learning in dogs´ acquisition of communicative skills [15, 16]. This perspective is based on the following facts: 1) puppies´ ability to follow human communicative cues, such as signaling, improves during development [17, 18]; 2) shelter dogs need longer training to learn to follow complex communicative cues than family dogs [19]; and 3) under some circumstances, wolves show better performance in communicative tasks than dogs [18].

In the present, most authors agree that both phylogeny and ontogeny play a fundamental role in explaining the development of domestic dogs´ communicative abilities with people [20, 21]. For example, Udell, Dorey, and Wynne (2009) proposed a two-stage hypothesis in which genetic dispositions as well as early interactions with people and life-time learning allow dogs to acquire their distinctive socio-cognitive and communicative abilities [16]. Nonetheless, there is still room for disagreement in terms of the relative contribution of each factor and how each contributes to the development and acquisition of these skills.

In this study, we focused on two important types of evidence that may contribute to this debate. First, we compared the performance of adult dogs with contrasting levels of everyday social experience with people as provided by the comparison between shelter and family dogs. And second, we used the same communicative task to assess the performance of puppies against that of adult dogs.

Dogs that spend long periods of time in canine shelters count on fewer opportunities to learn from people than those that live in homes as pets [19]. Moreover, it is common that shelter dogs have had more negative and even traumatic experiences with people [22, 23]. In terms of social skills, shelter dogs have shown important differences in their performance in communicative tasks as well as in their attitudes towards humans relative to family dogs [16, 21]. For example, Barrera, Mustaca, and Bentosela (2011) [24] compared the time family and shelter dogs spent looking at people in a task involving an out-of-reach reward, and found that shelter dogs persevered less in the gazing response in the extinction phase of this task.

Another way to assess the role of experience with people in dogs´ socio-cognitive performance is to evaluate puppies which, given their young age, have not had many opportunities to learn from humans. Recently, Zaine, Domeniconi, and Wynne (2015) [25] evaluated puppies of different ages and different levels of social contact (shelter dogs vs. pets) in diverse human signaling tasks. The amount of experience with people and age both contributed to improving their performance. Surprisingly, younger puppies with more experience with people outperformed older puppies raised in a shelter [25].

The goal of the present study was to investigate whether differential social stimulation contributed to dogs´ performance in a task in which subjects had to discriminate between generous and selfish people, defined as who gave or withheld food from the dog in a communicative context (a pointing task). To accomplish this goal, we trained dogs in an object-choice task in which an experimenter would make a pointing gesture towards a bowl in which food was hidden. Each dog went through two conditions. In training trials with the generous experimenter, this person allowed the dog to eat the food, whereas in training trials with the selfish experimenter, the person ate the food just before the dog could have access to it. After a training session with each experimenter, we ran a choice test in which both experimenters were simultaneously present and the dog was allowed to freely approach (choose between) them.

In brief, we compared the performance of adult family dogs against that of two other groups of dogs with lower quality and/or quantity of socialization with people: 1) adult shelter dogs; and 2) 45-60-days old puppies. If dogs require extensive experience with people to be able to discriminate human generous and selfish attitudes, we would expect puppies to show a poorer performance than that of adult pets. The prediction is not so clear relative to shelter dogs. On one hand, we could expect shelter dogs to present a lower performance given their reduced daily contact with people relative to adult family dogs. On the other hand, the fact that shelter dogs have usually gone through negative experiences with people [23] makes it likely that they pay more attention to human behavior and the consequences associated with it. In this last sense, the performance of shelter dogs in this communicative task could be expected to be at the same level or even higher to that of pets.

## Materials and methods

### Ethical statement

This study was carried out with the approval of the CICUAL (Institutional commission for the care and use of laboratory animals) from the medical research institute IDIM CONICET (Res. Nro. 012–14) and complied with the current Argentine law of animal protection (Law 14.346). All owners expressed their consent for the participation of their dogs in this study.

### Subjects

We evaluated three independent groups of dogs. The first group comprised family dogs living with their owners as pets since at least one year (group FD). The second group comprised dogs living in shelters for at least one year (group SHD). Dogs in groups FD and SHD were all mixed breed, and were 1–10 years old. The third group comprised puppies between 45 and 60 days old, of different breeds, and living in people´s homes (group PUP).

**Group FD:** We assessed 15 dogs; two of them were discarded from the sample because they showed signs of excessive fear. The final sample comprised 13 dogs (11 females and 2 males). Mean (±1 SD) age was 4.53 (2.04) years old. Eleven dogs were neutered and two were intact.

**Group SHD:** We evaluated 22 dogs living in the “Soplo de Vida” dogs’ shelter (http://www.soplodevida.org/) in the city of Merlo, Argentina. Three of them were discarded from the sample (one for excessive fear, one due to technical problems, and the other for lack of motivation to perform the task). The final sample comprised 19 dogs (10 females and 9 males), all of them neutered. Mean (±1 SD) age was 5.14 (2.55) years old. Shelter meals involved balanced food, once a day between 11.30 and 12.30 h. Pairs or lone dogs lived in 8 m^2^ kennels that allowed olfactory, auditory and visual contact with dogs in neighboring kennels. They also had a 450 m^2^ outdoor facility with grass and dirt in which they could walk (and run) for approximately two hours a day.

**Group PUP:** We evaluated 19 puppies. Four of them were discarded from the sample (one due to technical problems, two for lack of motivation, and one for showing signs of excessive of fear). The final sample comprised 15 puppies (5 females and 10 males) of different breeds (5 Dalmatian, 3 German Shepard, 3 English Bull Terrier, and 4 mix breed) from seven different litters. Mean (±1 SD) age was 50.53 (4.76) days old. All puppies lived at their owners’ homes since they were born and had daily contact with people.

### Apparatus

To test dogs’ ability to recognize human attitudes, we used an object choice task and a choice test. Three persons were needed for this protocol: a handler and two experimenters (hereafter, E or Es for the singular or plural forms, respectively), one performed the generous role (G), and the other performed the selfish role (S).

We differentiate between generous and selfish behavior as giving or withholding food from the dog in a communicative context (a pointing task). It is worth noting that although we operationally defined “selfish” and “generous” in relation to experimenters’ food sharing behavior, both the generous and the selfish experimenters acted as complex stimuli whose behavior also varied in other features such as their use of ostensive cues (see more details in the Methods section).

For the object choice task, we had two chairs, 75 cm apart from each other. On the seat of each chair, Es placed two identical opaque bowls (20 cm diameter and 8 cm height) to present the food to the subjects. For group PUP, we placed the bowls on the floor (we did not use chairs). To control for odor cues, we hid 5 pieces of food (baked chicken) under a double bottom in each bowl, and, besides, both bowls were greased with abundant chicken. During training trials in groups FD and SHD, an E stood between the chairs and a handler held the dog 1.5 m in front of the E. For the choice test, we took the chairs away, and G and S stood approximately where the chairs were.

We used tape to draw a 50 cm perimeter around each chair on the floor to delimitate the choice area during the object choice task and around each E in the choice test (see procedure below). A camera SONY DCR-SR88 was placed on a tripod behind and above the handler, and another camera (Sony DCR 308) was placed behind and above one of the chairs to video-tape the trials.

### Procedure

The procedure was similar to the one described in Carballo et al. (2015). It comprised a pre-training phase and two training phases. Each training phase comprised two training sessions, one with G and one with S, and a choice test (i.e., the whole protocol involved 2 training sessions with each E, and two choice tests in total). The inter-trial interval (ITI) was set at 20 sec, and intervals between sessions, and phases were set at 1 min.

We first describe the protocol used with adult dogs, and, then, we mention the changes implemented to evaluate subjects of group PUP.

**Pre-training:** the purpose of pre-training was to show dogs that the bowls could contain food. After a period of familiarization (3–5 min), G placed a bowl with food on the seat of each chair and left the scene. Then the handler led the animal towards each bowl and let the dog eat. This procedure was performed twice. Immediately after pre-training trials, the first training phase began.

**First training phase:** it comprised two sessions, one with G and the other with S, of six trials each. For half of the subjects, training began with G, and for the other half, it began with S; the same order was followed in the second training phase. For any given subject, G and S were experimenters of different gender (this eases the discrimination between them; see [2]). For half of the dogs, G was a woman and S a man, and, for the other half, it was the other way round.

In the beginning of any training trial, out of the dog’s sight, the corresponding E (G or S) hid a piece of food in one of the two bowls. Then, the E took her/his position in between the chairs, and simultaneously placed each bowl on the seat of a chair. The side in which the bowl with food was placed (left or right) was randomized in each trial with the restriction that we did not use the same side in more than two consecutive trials. Afterwards, the E did the pointing gesture towards the bowl with food, and the dog was released to find the reward. We used a static proximal pointing gesture as signaling cue [26].

In training trials with G, before the pointing gesture, the E looked at the dog’s face and called it by its name in a positive manner until catching its attention. Then, G did the pointing gesture, while also giving ostensive cues: alternating the gaze between the dog and the food bowl and saying twice in Spanish “Mmm… Look inside, it’s so yummy!” using a positive intonation. The handler then released the dog. If the dog chose the baited bowl, it was allowed to eat the food in it. If the dog chose the empty bowl, G pointed again towards the correct bowl until the dog approached it and ate the food (i.e., the dog always ate from the bowl in training trials with G). While it was eating, the dog was verbally reinforced by G who said in Spanish “Very good!”, though Es never initiated any physical contact with the dog. After the dog ate, the handler took the subject to the starting position again, and the following trial with G began. Once all six trials with G were done, the session with S or the choice test began.

In training trials with S, once the E was in between the chairs and with the bowls in place, S looked at the dog and called it by its name in a neutral tone of voice until catching its attention (a maximum of 3 calls). Then, S would make the pointing gesture towards the bowl with food without saying anything and while directing her/his gaze towards the signaled bowl. The handler then released the dog and, if it approached the correct bowl, S quickly took the food from the bowl, showed it to the animal, ate it in front of it, and let the subject see the empty bowl. If the dog chose the empty bowl, S did the pointing gesture towards the correct bowl again until the dog chose it and then S ate the food in front of the dog as described. After that, the handler led the dog to the starting position again, gave the subject a piece of food (to equalize rewards delivered in sessions with G and S), and the following trial with S began after 20 sec. Once the six trials with S were done, there was a 1-min interval until either the session with G or the choice test began.

In the case a dog did not approach any bowl within 15 sec since the beginning of the pointing gesture in any given training trial, we registered a “no-choice” response. After a no-choice response, the handler gently led the subject towards the E, thus encouraging the dog to choose in order to be exposed to the corresponding E’s attitude and subsequent food reward.

**First choice test:** one min after the end of the first training phase (i.e. after a session with G and a session with S), we ran a choice test. Chairs were removed, and G and S stood approximately where the chairs had been (though a bit further apart, at a distance of 1.5 m), each holding a bowl at the chest level, and looking at the dog. Once both Es were in position, the handler held the dog at the starting position for about 2 s to make sure that the animal had time to observe the scene, and then, she released the subject to allow it to approach and choose between the Es.

We considered a “choice response” when the dog approached one of the Es with its head facing him/her at distance of less than 50 cm according to the tape marks on the floor. If the dog did not make a choice within 15 sec since released, we registered a “no-choice” response. No reinforcement was delivered during the choice test. The side in which G and S stood in the choice test (left or right) was counterbalanced between the first and the second choice test for each dog, and also between subjects.

**Second training phase and second choice test:** One min after the end of the first choice test, the second training phase began which was exactly the same as the first one. It was also followed by a choice test which was exactly the same as the first choice test, with the exception that G and S stood on opposite sides relative to those occupied in the first test.

For group PUP, we introduced the following changes in order to adapt the protocol to their possibilities. First, the bowls were placed on the floor, instead of on the chairs seats. Second, the Es kneeled down to give the communicative cues. Third, given that the main goal of our study was to evaluate puppies’ abilities to discriminate human generous and selfish attitudes, not to evaluate their skills for following pointing gestures, we used a simpler communicative cue, namely pointing plus body position. That is, at the beginning of any training trial, the E stood in front of the dog, kneeled down and placed both bowls simultaneously on the floor at a distance of approximately 1 m. Then, she/he kneeled behind the baited bowl, called the puppy by its name, and gave the pointing gesture with her/his ipsilateral arm. In choice tests, Es kneeled on the floor (an E on the left and the other on the right), each holding a bowl at the chest level. The rest of the protocol continued as described for adult dogs.

### Data analyses

We measured five dependent variables (further explained in the paragraph below): In training trials, we measured the latency to choose a bowl (latency) and the number of correct, incorrect and no choices (performance). In choice tests, we measured dogs’ first choice (choice), the time spent in proximity to each E (proximity), and the time spent looking at each E (gaze). All measures were taken from the videos using SONY Vegas 11 Pro video Editor. To determine a dog’s first choice, the time in proximity, and the time spent looking at each E in choice tests, we did a frame by frame analysis which involved watching 3 frames per second. Therefore, given that choice tests lasted for 15 sec, we analyzed 45 fixed frames per test.

**Latency** was defined as the time (sec) elapsed since the E gave the pointing gesture until the moment the dog had its nose at 10 cm from any bowl. If dogs did not choose any of the bowls, we registered a15sec latency. In 20% of training trails, latencies were also measured by a second rater. Correlation between raters’ measures were very high (Pearson correlation coefficient: FD: *r* = 0.95, *P* < 0.001; SHD: *r* = 0.95, *P* < 0.001; PUP: *r* = 0.99, *P* < 0.001). Given that latencies were not normally distributed, we log-transformed them to be able to use parametric tests. After transforming latencies, their distribution did not significantly differ from a normal distribution (Kolmogorov-Smirnov test: all *P*-values > 0.1). To ease interpretation though, latencies are reported in sec in the text and table. To analyze the variation in latencies across trials and among groups, we ran repeated measures ANOVAs with training phase (1 and 2), trial number (1 to 6 and 7 to 12) and Es’ attitude (G vs S) as within-subject factors, and group (FD, SHD, PUP) as between-subject factor. We also included the size and sex of the dogs, the order in which training began (first G or S), and the sex of the E as between-subject factors to control for their possible effects. None of these factors had a significant effect on latencies (all p-values>0.05). Thus, we re-ran and report the analysis without including these factors.

**Performance:** during training trails we registered whether dogs chose the pointed bowl (i.e., the bowl with food inside; coded as a “correct response”), the other bowl (coded as “incorrect response”), or did not make a choice within 15 sec of the pointing gesture (coded as “no response”). We compared the number of correct responses against the 0.5 chance level in each group using *t*-tests. We also compared the number of correct, incorrect, and no responses using ANOVAs with group as a between-subject factor, and Es’ attitude (G or S) as a within-subject factor. We also analyzed the performance of each group separately comparing the number of correct, incorrect, and no responses between trials with G and with S using paired *t*-tests.

**Choices:** in choice tests, we registered which E the dogs first approached. If dogs did not approach any E during the 15 sec of the choice trial we coded a “no choice” response. All choices were independently codified by two of the authors (FC and MB); in cases of disagreement, we used the evaluation of an assistant who was unfamiliar with the experimental design. To evaluate dogs’ choices, we ran a GLMM with choice as a binomial dependent variable with a logit linking function. Dogs that did not choose were removed from the analysis. We included the group (PD, SHD, PUP), the order of training session (first G then S or vice versa), and the gender of the Es as fixed factors, and the id, sex and size (big vs medium/small) of the dogs as random effects. The number of test (first or second) was included as repeated measure. None of the complementary factors showed a significant influence on the probability of dogs choosing G or S (all p-values > 0.05). Therefore, we re-ran the analysis with only subject id as random factor, group as a fixed factor, and test as a repeated measure. We used the estimated parameters of this last model (because it was more parsimonious and have a smaller AIK and BIC criterion) for comparisons between groups and to test dogs´ choices against chance level (0.5).

Additionally, from the frame by frame data of choice trials, we counted the number of frames in which dogs were within 50 cm of each E according to the marks on the floor (**Proximity**), and the number of frames in which dogs were looking at each E (**Gaze)**. A second rater also measured 20% of the data. Correlations between raters’ measures were very high (for both measures–proximity and gaze- and the three groups–FD, SHD, and PUP-, all *r*-values were between 0.93 and 0.99, all *P*-values<0.001). As these measures were not normally distributed, we used the Kruskal-Wallis test to compare the time spent in proximity and gazing to each E among groups. To compare the time spent near and looking at G and S in each group, we used Wilcoxon tests.

The α-value was set at 0.05 and all tests were two tailed. We used SPSS statistical package v. 19 for the analyses.

## Results

### Training phase

Table 1 shows the main results (latencies, performance, and choices). Raw data is available in S1 Data.

**Table 1:** Mean (SD) latency (sec), proportion of choices of the pointed bowl in training sessions, and proportion of choices for G over S in first and second choice tests.

		Latencies		Proportion of correct choices		Proportion of choices to G Over S	
		Training Phase		Training Phase		Test	
Group	Experimenter	First	Second	First	Second	First	Second
**FD**	G	2.81 (2.18)	2.10 (0.74)	0.82	0.87	0.76	0.61
	S	2.95 (1.13)	3.26 (1.78)	0.83	0.85		
**SHD**	G	3.36 (1.92)	3.16 (1.23)	0.83	0.83	0.78	0.58
	S	4.78 (2.93)	4.87 (3.27)	0.73	0.71		
**PUP**	G	2.83 (2.01)	3.04 (3.00)	0.83	0.82	0.53	0.70
	S	2.84 (1.74)	3.74 (2.88)	0.80	0.77		

FD: adult family dogs; SHD: adult shelter dogs; PUP: puppies. G: generous experimenter; S: selfish experimenter.

**Latencies:** We found a main effect of Es’ attitude (F_1, 41_ = 13.49, *P* = 0.001). Dogs took longer to approach the bowl pointed by S (M = 3.84, SE = 0.34) than by G (M = 2.97, SE = 0.23), whereas we found no effect of group (F_1, 41_ = 1.92, *P* = 0.15), or of the E’s attitude x group interaction (F_2, 41_ = 0.68, *P* = 0.51). We found an effect of the interaction of trial number x Es’ attitude (F_5, 205_ = 6.47, *P* <0.001). Latencies towards S increased, whereas latencies towards G decreased across trials. No other significant differences were found (all *P*-values > 0.05).

Analyzing the latencies of each group separately, we found that subjects in groups FD and SHD took longer to approach S than G (FD: F_1, 11_ = 7.52, *P* = 0.019; SHD: F_1, 18_ = 7.62, *P* = 0.013), whereas puppies did not (F_1, 12_ = 1.35, *P* = 0.26). We also observed an interaction effect between trial number and Es’ attitude in both adult groups (FD: F_5, 55_ = 5.93, *P* <0.001; SHD: F_5, 90_ = 4.95, *P* <0.01), though not in group PUP (F_5, 60_ = 0.77, *P* = 0.57). No other significant effect was found.

**Performance:** Dogs in group FD chose the pointed bowl in 84.6% of trials with both Es, whereas dogs in group SHD chose the pointed bowl in 83.3% of trials with G and in 72.3% of trials with S. Subjects in group PUP chose the pointed bowl in 82.7% of trials with G and in 78.8% of trials with S. Dogs in all three groups followed the pointing gesture above the chance level with both Es (*t*-test: all *P*-values < 0.01).

Comparing the performance among groups showed no differences in the number of correct or incorrect responses between Es (G vs. S). No interaction effects were detected between groups and Es’ attitude (ANOVA, all *P*-values > 0.05). Nevertheless, we found a main effect of Es’ attitude (F_1, 44_ = 9.53, *P* = 0.003), and group x Es’ attitude interaction (F_2, 44_ = 4.75, *P* = 0.014) in the number of no responses. Dogs in group SHD showed more no responses than dogs in groups FD and PUP. In addition, there were more no responses in trials with S than in trials with G.

When we analyzed the performance of each group separately, we found no differences in the number of correct, incorrect and no responses between trials with G and S in both family groups (FD and PUP: *t*-test: both *P*-values > 0.40). Dogs in group SHD showed less correct responses with S than with G (*t*_18_ = 2.4, *P* = 0.027), and also more no responses with S than with G (*t*_18_ = -4.05, *P* = 0.001). The number of incorrect choices was not influenced by Es’ attitude (*t*_18_ = 0.29, *P* = 0.77).

### Choice tests

**Fig 1:**
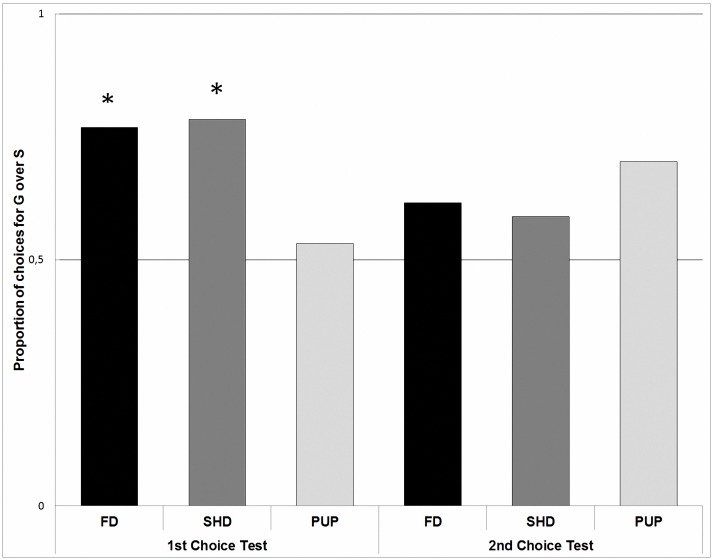
Proportion of choices for the generous (G) over the selfish (S) experimenter in the first and second choice tests as a function of group (FD: adult family dogs; SHD: adult shelter dogs; and PUP: puppies).

**Choice:** Fig 1 shows the proportion of choices for G over S in both choices tests. In the first choice test, 10 dogs out of 13 chose G, and 3 chose S in group FD. In group SHD, 11 dogs out of 19 chose G, 3 chose S, and 5 did not choose. In group PUP, 8 dogs out of 15 chose G, and 7 chose S. In the second choice test, 8 dogs out of 13 chose G, and 5 chose S in group FD. In group SHD, 10 dogs out of 19 chose G, 7 chose S, and 2 did not choose. Finally, in group PUP, 7 dogs out of 15 chose G, 3 chose S, and 5 did not choose.

We did not find any main effect of group, or test phase in the probability of choosing G over S (all p-values > 0.05). When we compared the proportion of choices against the 0.5 chance level, we found a preference for G over S (*t*_81_ = -2.95, *P* = 0.004). Comparing the performance of all groups together in each test separately, we found that dogs preferred G over S only in the first choice test (*t*_41_ = -3.34, *P* = 0.001), but not in the second (*t*_39_ = -1.43, *P* = 0.16). Nevertheless, there were no significant differences between choice tests in the choices for G over S (F_1, 76_ = 1.21, *P* = 0.27). Analyzing the first choice in both test sessions of each group, only SHD choose the G over S above chance level (*t*_30_ = -2.44, *P* = 0.01).

Finally, comparing the first choice of each group against chance in each test separately, we found that only FD (*t*_12_ = -2.72, *P* = 0.02) and SHD (*t*_13_ = -3.55, *P* = 0.003) showed a preference for G over S in the first test, whereas PUP chose at chance level (*t*_14_ = -0.92, *P* = 0.36). In the second test, subjects from none of the groups showed a clear preference for G or S (all p-values > 0.05).

**Proximity:** In the first choice test, dogs in group FD spent a mean (±1SD) of 8.41 ± 5.35 sec near G, and 5.33 ± 5.60 sec near S. Dogs in group SHD spent 6.28 ± 5.50 sec near G, and 1.49 ± 2.47 sec near S. Dogs in group PUP spent 6.30 ± 5.09 sec near G, and 5.47 ± 6.11 sec near S. We found group differences in the time dogs spent near S (Kruskal-Wallis: *H*_*2*_ = 5.47, *P* = 0.05). Dogs in group SHD spent less time near S than dogs in groups FD and PUP. No other differences between groups were found. Moreover, subjects in group SHD spent significantly less time near S than near G (Wilkoxon: *Z* = -2.64, *P* = 0.008). The comparison between the time spent near each E was not different in groups FD (*Z* = -1.1, *P* = 0.23) and PUP (*Z* = -0.48, *P* = 0.62).

In the second choice test, dogs in group FD spent 6.73 ± 4.80 sec near G, and 5.53 ± 4.60 sec near S. Dogs in group SHD spent 6.26 ± 5.41 sec near G, and 3.01 ± 3.63 sec near S, and dogs in group PUP spent 6.00 ± 4.32 sec near G, and 3.52 ± 3.37 sec near S. We did not find group differences in the time dogs spent near S or G (G: *H*_*2*_ = 0.23, *P* = 0.89; S: *H*_*2*_ = 3.52, *P* = 0.16). When we analyzed each group separately, we did not find any differences in the time spent near each E in any group (all *P* -values > 0.05).

**Gaze:** In the first choice test, dogs in group FD gazed for 6.92 ± 4.32 sec at G, and for 3.61 ± 3.38 sec at S. Dogs in group SHD gazed for 4.29 ± 3.51 sec at G, and for 1.49 ± 2.47 sec at S, and dogs in group PUP gazed for 2.61 ± 3.16 sec at G, and for 2.09 ± 2.54 sec at S. Dogs in group FD looked longer at G than dogs in group PUP, but not more than dogs in group SHD (*H*_*2*_ = 9.20, *P* = 0.009). No differences among groups were found in the time looking at S (*H*_*2*_ = 5.00, *P* = 0.08) or in the time looking at each E when assessed in each group separately (all *P* -values >0.05).

In the second choice test, dogs in group FD gazed for 4.48 ± 3.77 sec at G, and for 4.94 ± 2.66 sec at S. Dogs in group SHD gazed for 4.73 ± 4.09 sec at G, and for 2.38 ± 1.72 sec at S, and dogs in group PUP gazed for 2.26 ± 2.50 sec at G, and for 1.21 ± 1.79 sec at S. Dogs in group FD looked longer at S than dogs in groups SHD and PUP (*H*_*2*_ = 15.8, *P* <0.001). No difference among groups were found in the time spent looking at G (*H*_*2*_ = 3.52, *P* = 0.16). When we analyzed each group separately, we did not find any differences in the time spent looking at each E (all *P* -values >0.05).

## Discussion

The discrimination of people based on their past behavior is essential to domestic dogs [27] given that most relevant resources for these animals -such as, food, water, and shelter are provided by humans [28, 16].

In the present study, we sought to compare the performance of dogs with different degrees of social experience with humans in a discrimination task of human attitudes. To this end, the behavior of adult family dogs (FD) was contrasted against that of adult shelter dogs (SHD) and that of 45-60-days-old puppies (PUP). Shelter dogs have less everyday-contact with people than family dogs, whereas puppies, though they have everyday contact with their owners at home, have had fewer opportunities than adult dogs to learn from interactions with people because of their young age. Dogs of all groups interacted with a generous (G) and a selfish (S) experimenter. In training sessions, G allowed the dog to eat from a bowl with food, whereas S ate the food herself, and therefore did not allow the dog to eat from the bowl. In choice tests, subjects could freely choose to approach G and S.

To begin with, we found that both groups of adult dogs (FD and SHD) preferred to approach G over S in the first choice test. In contrast, puppies did not show this preference, and their choices between G and S did not differ from random in the first or second choice test. This difference between adult dogs and puppies could reflect differences in their capacity to discriminate and remember the experimenters´ differential attitudes.

Dogs´ latencies in training trials provide further evidence that adult dogs learned to discriminate between G and S, whereas puppies did not. Adult dogs increased their latencies to approach S across trials, but puppies did not show any evidence of change in the time to approach G or S throughout training. Though these latencies could be taken to suggest an increased discrimination between G and S, subjects did not show any significant preference between G and S in the second choice test. This lack of bias in choices, despite latencies showing otherwise, could be explained by the fact that dogs were not reinforced in the first choice test, and thus, the second choice could have resulted more ambiguous to them. In fact, this pattern of choice results (a clear preference in the first choice test that disappears in the second choice test) coincides with previous studies [2].

In short, the data considered up to this point suggest the dogs´ capacity to discriminate between human generous and selfish attitudes, as tested in the present protocol, does not depend so much on the quality of everyday contact with people, but more so on the amount of experience with humans (in years). This conclusion would, however, imply that shelter dogs behaved exactly the same as adult family dogs, which was not the case.

In the present study, shelter dogs presented more “no responses” to the pointing gesture, and thus, had fewer correct responses than subjects in group FD. This result could be due to two non-exclusive processes. First, dogs of group SHD could have been more fearful towards strangers, avoid approaching the Es, and thus taking longer to learn the association between G and the bowl with food. This interpretation is consistent with a study showing that shelter dogs took longer to learn to follow human complex communicative cues (momentaneous distal pointing) than family dogs [19]). Second, the other potentially involved process behind “no responses” could be punishment. From this perspective, while the animal develops the discrimination between G and S, it learns that following G’s pointing is associated with obtaining of an appetitive reinforcer (the food) and following S’s pointing leads to a negative consequence (food being eaten in front of the subject). In punishment protocols, an animal diminishes the response rate after experiencing negative consequences of its behavior [29]. Taking into account that most of the no responses were observed in trials with S, it is possible that this behavior means that subjects from group SHD were more sensitive to punishment than FD. Furthermore, given that in trails with no responses a 15 sec maximum latency was registered, more no responses in group SHD led to longer latencies in that group relative to FD. This pattern of behavior in group SHD is consistent with the idea that those subjects (SHD) are more sensitive to the negative consequences of their interactions with humans than dogs from group FD.

Furthermore, we found that adult family dogs spent more time watching the experimenters in choice tests than shelter dogs. Miklósi, Kubinyi, Topál, Gácsi, Virányi, and Csányi (2003) [30] have shown that family dogs gaze towards people as a request for help. Consistent with the present evidence, this type of begging behavior has been shown to be modulated by dogs´ short- and long-term history of learning with people [24]. For instance, family and shelter dogs reinforced to direct their gaze towards the experimenter showed differential rates of extinction; more specifically, family dogs took longer to extinguish such behavior [24].

In choice tests, we measured the time dogs spent gazing and close to the Es as indirect measures of dogs’ preference between G and S. Nevertheless, these behaviors may be driven by different motivations. As discussed previously, gazing behavior could be interpreted as a way of soliciting help from a person [30], whereas proximity could be driven by the desire of social contact [31]. Indeed, previous research showed that pet dogs took longer to extinguish their gazing behavior towards humans when a reward was unavailable compared to shelter dogs [25], whereas shelter dog spent more time near an unknown human in a sociality task [31]. These results could be interpreted as if pet dogs were more resistant to extinguishing a previously learnt communicative response that may lead to obtaining a reward [32], possibly due to their presumed life-long history of previous reinforcement from [33, 34]. Meanwhile shelter dogs could be more motivated to look for human contact (i.e. [35]). Evidence consistent with this interpretation comes from Barrera et al., (2015) study in which dogs could interact with an apparatus to get food in the presence of an experimenter or in a lone condition. Shelter dogs look longer and stayed closer to the E than pet dogs which, in turn, stayed longer interacting with the apparatus. Pet dogs´ resistance to extinction was also observed in the lone condition. This might indicate that the person is a stronger stimulus for shelter dogs, and that pet dogs are more perseverant in the reward seeking behaviors [32–33].

The described differences in performance between family and shelter dogs may then depend on diverse possible causes. First, these differences could be suggestive of SHD subjects needing more time to habituate to the experimenters given their reduced everyday contact with people. Second, shelter dogs usually have fewer opportunities to associate people with rewards than family dogs, which could have facilitated the latter´s association of person and reward [33]. Last but not least, it is common that shelter dogs have been through situations of maltreatment and abandonment [22]. This could make them more fearful towards strangers [23], such as the experimenters in the present protocol. All in all, behavioral differences between adult family dogs and shelter dogs in the present study could respond to both/either the cumulative positive experiences that family dogs enjoy with their owners and other people, and/or the shelter dogs´ history of negative experiences with humans. The present study does not allow discriminating between these two possibilities. Despite these differences, it is worth remembering that both family and shelter dogs developed a preference for the generous over the selfish experimenter in the first choice test.

A methodological issue regarding group SHD, however, limits the conclusions of our findings. We did not count on information about the origin of dogs in group SHD. They could have been former stray or pet dogs [36]. Thus, it is possible that a year was not sufficient time for former pet dogs (turned into shelter dogs) to unlearn their responses to humans. Nevertheless, this limitation regarding the previous history of shelter dogs is a common factor in most of the studies assessing shelter dogs and, besides, many previous studies have nonetheless found differences between family and shelter dogs, even relying on shelter dogs that had spent less than a year in the shelter environment [24–25, 35].

To finish, we want to go back to the theoretical perspectives mentioned in the introduction, that is, the dispute between the role of domestication and learning in the development of dogs´ socio-cognitive abilities (see references [13–16]). This dispute has a parallel in discussions over the development of human social cognition. For instance, Hamlin, Wynn, and Bloom (2007) [37] made babies of 6–10 months witness scenes in which a character allowed or did not allow another character to climb a mountain, and found that subjects developed a preference for the allowing than the impeding character. Whereas Hamlin et al., (2007) [37] concluded that this evidence suggested that babies have an innate capacity for moral evaluation, Scarf, Imuta, Colombo, and Hayne (2012) [38] could not replicate these results when they controlled for the climbing character´s reaction to the allowing or impeding behavior. This result led Scarf et al., (2012) to conclude that attributing moral evaluation to babies was unwarranted [38]. Complex behaviors develop from simpler elements, which can be detected before the full-fledged pattern can be identified.

Similarly, present data cannot unequivocally support the claim that dogs have an innate communicative ability through which they manage to interact successfully with people. The fact that adult subjects, both pets and shelter dogs, showed a preference for the generous person suggests that the amount and quality of everyday experience with people is not essential to learn a proficient discrimination of contrasting human attitudes. Since we do not have data about the early experience of shelter dogs, we cannot be certain whether their experience with people in their critical socialization period as puppies was different from that of pets. If that were the case, the implications would be that dogs may not need rich social experience with people to develop the socio-cognitive skills tested in the present task.

Another limitation of the present study is that it does not allow distinguishing whether puppies´ differential performance and choices respond to insufficient experience with people or lack of maturation of relevant processes. Despite this, we believe that this last interpretation (lack of maturation) is unlikely given the following reasons.

First, the protocol used in this study is similar to a discrimination learning task in which the presence of a given stimulus (G) indicates the availability of a reward, whereas another stimulus (S) signals the absence of an immediate reward. Then, the choice test allows checking whether subjects learned the discrimination. There is evidence that puppies, as young as 3 days old, are capable of learning the discrimination between a conditioned stimulus that anticipates the availability of milk and another stimulus that anticipates an aversive drink or no reward [39, 40]. This shows that puppies can discriminate between stimuli with differential predictive value from a younger age than that of the puppies tested in the present study. In the present protocol, however, puppies were unsuccessful in achieving the discrimination between G and S probably because these discriminative stimuli were too complex (i.e., with many features) and involved many subtleties such as gestural, verbal, and attitudinal cues.

Second, the ability to discriminate differential human attitudes from different individuals and later choose between them based on that discrimination implies a process of individual recognition of the actors involved [2, 41]. Domestic dogs can readily recognize familiar people [42], but little is known about their capacity to discriminate among unknown persons or how much training is required to develop such discriminations. To the present, most evidence suggests that individual recognition is based, at least partly, on basic conditioning processes [2, 43], thus stressing the role of experience in the development of this ability.

Third, the motor skills of puppies of the selected age range (45 to 60 days old) should have been fully developed already [44]. Therefore, it seems unlikely that differences between puppies and adult dogs were due to motor immaturity in the former group. In sum, given that dogs´ discrimination of verbal and gestural cues from people and recognition of individuals rely on everyday experience and accumulated learning, we think that puppies in the present study were incapable of solving the discrimination task at hand because of lack of experience with people (and strangers in particular) more than because of immaturity. In any case, more studies are needed to achieve more definitive answers to these questions.

To conclude, we argue that present data would be consistent with Udell et al.´s (2009) two-stage hypothesis which posits that dogs´ sensitivity to human communicative cues depends on two types of experiences [16]. First, dogs must have contact with people during their socialization sensitive period. This leads to the development of dogs´ acceptance of people as social partners. Indeed, some authors argue that the domestication process has lengthened this sensitive period [45]. Second, the possibility of living in human environments presents dogs with plenty of opportunities to learn to predict and get relevant information from human behavior. All in all, both critical exposure to people and life-time learning in human environments allows dogs to display sophisticated socio-cognitive skills in their interaction with people.

## Supporting information

S1 Data Supporting data. (XLSX)
